# Supplementary material for: Probing bacterial cell wall growth by tracing wall-anchored protein complexes
Source: Nat Commun. 2021 Apr 12;12:2160. doi: 10.1038/s41467-021-22483-8 (PMC8042023; doi:10.1038/s41467-021-22483-8)
Supplement: Supplementary file 1 — Supplementary Information [file 41467_2021_22483_MOESM1_ESM.pdf]

Supplementary Information:

Probing bacterial cell wall growth by tracing wall-anchored protein complexes

Yi-Jen Sun[1]†, Fan Bai[2]†, An-Chi Luo[1], Xiang-Yu Zhuang[1], Tsai-Shun Lin[1], Yu-Cheng Sung[1], Yu-Ling Shih[3], Chien-Jung Lo[1]*

[1]Department of Physics and Graduate Institute of Biophysics, National Central University, Jhongli, Taiwan 32001, ROC

[2]Biomedical Pioneering Innovation Center (BIOPIC), School of Life Sciences, Peking University, Beijing 100871, China

[3]Institute of Biological Chemistry, Academia Sinica, Taipei 115, Taiwan, ROC

*Correspondence to: Chien-Jung Lo cjlo@phy.ncu.edu.tw; †These authors contributed equally to this work

Supplementary Figures

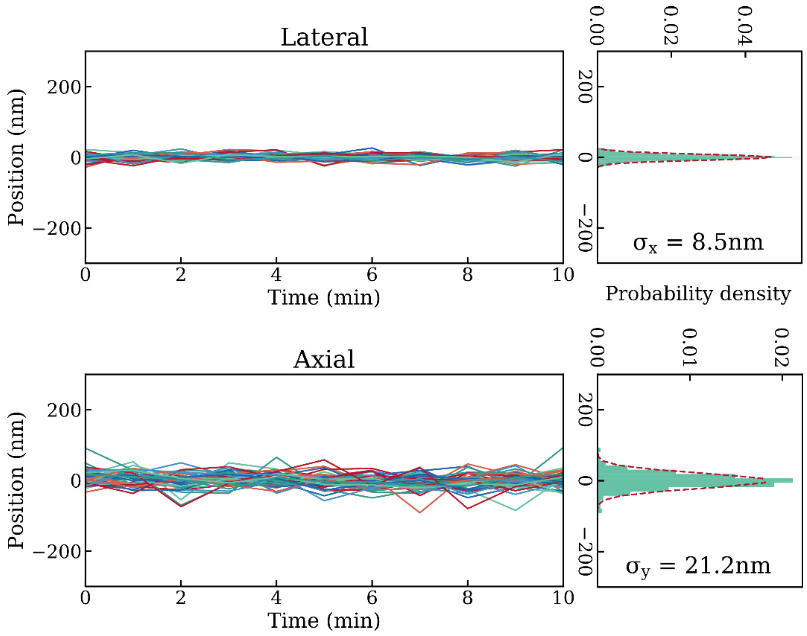

Supplementary Fig.1. Stability of the BFM anchoring position in live non-growing cells. BFM positional traces over 10 min in the buffer solutions (n = 48). The standard deviation of the lateral and axial coordinates was 8.5 nm and 21.2 nm, respectively.

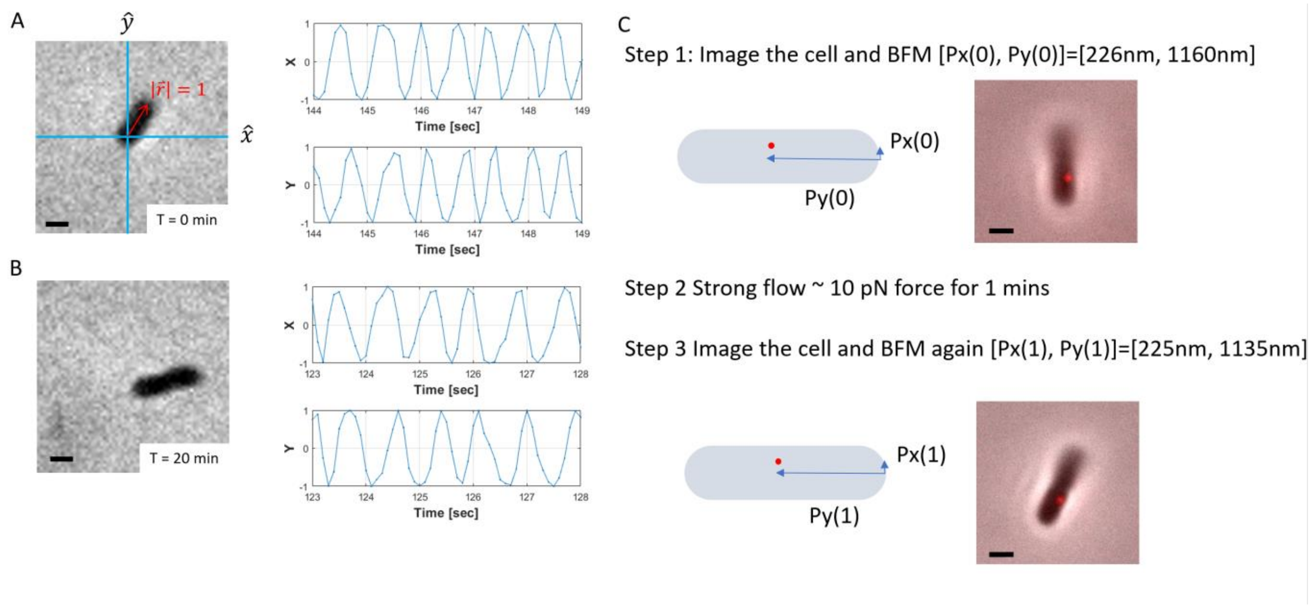

Supplementary Fig. 2. Additional experimental results to support that BFMs are firmly anchored to the cell wall during cell elongation. (A) A tethered cell experiment showing stable rotation of *E. coli* cell body in growth medium. (Scale bar, 1 μm) (B) The same cell as in (A) showing stable rotation of cell body after 20 min in growth medium while the cell elongates. (Scale bar, 1 μm) (C) A tethered cell rotated by a BFM and the hook of the BFM was fluorescently labelled. The coordinate of the BFM from the cell pole was [226 nm, 1160 nm]. After a strong hydrodynamic flow, the BFM position from the cell pole was [225 nm, 1135 nm]. (Scale bar, 1 μm).

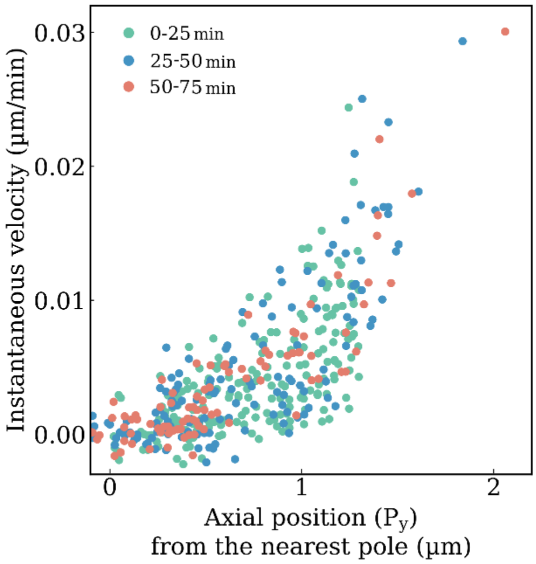

Supplementary Fig. 3 The relationship between instantaneous velocity of BFM movement during cell growth. The measurement time interval is 25 min. Green dots: 0-25 min; blue dots: 25-50 min; red dots: 50-75 min.

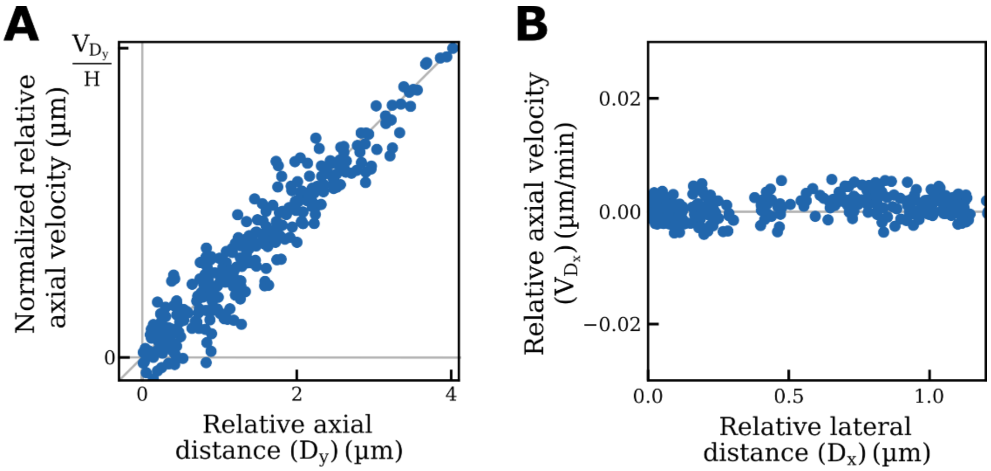

Supplementary Fig. 4. (A) Normalized BFM relative axial velocity vs. relative axial distance has a linear relationship, n = 63 (number of datasets). (B) All BFM lateral velocities from 62 datasets showed zero growth rate.

Strain	MTB9	*J. Bacteriol.* **194**, 3495–3501 (2012)	SiteC_flgE
Plasmid	pWR20	*PLoS One*. **7**, 1–10 (2012)<br>*Biophys. J.* **117** (2019).	Reference for plasmid pWR20
Primer	FlgE_seq	5’ GGTGTGATCCGCGGCAACAG 3’<br>5’ CCTGTTGATTCAGTGTCTGG 3’	FlgE primers dedicated for sequencing
Primer	pWR20 backbone	5’ AAAGCGGCCGCGGTGATTGATTGAGCAAG 3’<br>5’ AAACCTAGGATGTATATCTCCTTAACTAGGT 3’	

Supplementary Table 1. Strain, plasmid and primers information
