# Supplementary material for: Probing bacterial cell wall growth by tracing wall-anchored protein complexes
Source: Nat Commun. 2021 Apr 12;12:2160. doi: 10.1038/s41467-021-22483-8 (PMC8042023; doi:10.1038/s41467-021-22483-8)
Supplement: Supplementary file 3 — Description of Additional Supplementary Files [file 41467_2021_22483_MOESM3_ESM.pdf]

Description of Additional Supplementary Files

File Name: Supplementary Software 1
Description: All custom-written Python scripts for imaging and data analysis are provided in the supplementary software.
