# Supplementary material for: Probing bacterial cell wall growth by tracing wall-anchored protein complexes
Source: Nat Commun. 2021 Apr 12;12:2160. doi: 10.1038/s41467-021-22483-8 (PMC8042023; doi:10.1038/s41467-021-22483-8)
Supplement: Supplementary file 4 — Supplementary Software 1 [file 41467_2021_22483_MOESM4_ESM.zip › code-20210302.docx]

- Quantifying the size of inert zone (Figure 1C,1D)

from skimage import io

import numpy as np

import scipy as sp

import matplotlib.pyplot as plt

#from scipy.optimize import curve_fit

import os

from scipy import interpolate

import glob

from scipy.optimize import curve_fit

import matplotlib as mpl

from cycler import cycler

import pandas as pd

from pandas.core.frame import DataFrame

mpl.rcParams['axes.linewidth'] = 1.5

label_size = 20

mpl.rcParams['xtick.labelsize'] = label_size

mpl.rcParams['ytick.labelsize'] = label_size

mpl.rcParams["font.family"] = 'serif'

#--------------------------------------------------------------

# Get current size

fig_size = plt.rcParams["figure.figsize"]

# Prints: [8.0, 6.0]

print("Current size:", fig_size)

# Set figure width to 12 and height to 9

fig_size[0] = 16

fig_size[1] = 7

plt.rcParams["figure.figsize"] = fig_size

#-------------------------------------------------------------

def piecewise_linear(x, x0, k2):

return np.piecewise(x, [x < x0], [lambda x: 0, lambda x: k2*(x-x0)])

def fsigmoid(x, a, b, c):

return 1.0 / (1.0 + np.exp(-a*(x-b)))

def fitf(x, a, b): # this is your 'straight line' y=f(x)

return a*x + b

def onclick(event):

global ix, iy

ix, iy = event.xdata, event.ydata

#print ('x = %d, y = %d'%(ix, iy))

global xcoords,ycoords

xcoords.append(ix)

ycoords.append(iy)

if len(xcoords) == 1:

fig.canvas.mpl_disconnect(cid)

plt.close(fig)

return xcoords,ycoords

def __d(p1,p2):

dd = np.sqrt((p1[0]-p2[0])**2+(p1[1]-p2[1])**2)

return dd

def __ind(x,y,s):

temp = 10000

p1 = np.zeros((2,))

p1[0] = x

p1[1] = y

for k in range(0,len(s)):

d = __d(s[k,:],p1)

if(d < temp):

ind = k

temp = d

return ind

def __spline(data,num_pt):

x = data[:,0]

y = data[:,1]

# get the cumulative distance along the contour

dist = np.sqrt((x[:-1] - x[1:])**2 + (y[:-1] - y[1:])**2)

dist_along = np.concatenate(([0], dist.cumsum()))

# build a spline representation of the contour

spline, u = interpolate.splprep([x, y], u=dist_along, s=0)

# resample it at smaller distance intervals

interp_d = np.linspace(dist_along[0], dist_along[-1], num_pt)

interp_x, interp_y = interpolate.splev(interp_d, spline)

return np.array([interp_x,interp_y]).transpose()

#------------------------------------------------------

fig = plt.figure(0)

ax1=fig.add_subplot(1,2,1)

ax2=fig.add_subplot(1,2,2)

plt.subplots_adjust(left=0.07, right=0.97, top=0.9, bottom=0.1)

resize = 2

x100 = 13/250

path = "D:\\desktop\\Tracking ver.5"

os.chdir(path)

#---------------------------------------------------------------------

df = []

motorname = [["0802","1-2_5","5"],

["0802","1-3_2","5"],

["0802","2-1_2","5"],

["0802","2-1_3","5"],

["0802","3-1_1","5"],

["0802","3-2_1","5"],

["0802","3-2_4","5"],

["0802","3-3_1","5"],

["0802","4-3_1","5"],

["0802","5-1_2","5"],

["0802","7-1_1","5"],

["0802","8-1_1","5"],

["0815","4-1_2","5"],

["0815","4-1_5","5"],

["0824","2-1_1","2.5"],

["0824","2-2_1","2.5"],

["0725","1-1_2","5"],

["0725","1-2_1","5"],

["0725","2-1_2","5"],

["0725","3-1_4","5"],

["0725","3-2_1","5"],

["0725","4-2_3","5"],

["0725","6-4_1","5"],

["0725","7-1_2","5"],

["0725","8-2_1","5"],

["0725","9-1_1","5"],

["0725","10-1_4","5"],

["0810","3-1_1","5"],

["0810","5-1_1","5"],

["0810","6-1_1","5"],

["0810","6-1_2","5"],

["0803","2-1_2","5"],

["0803","3-1_3","5"],

["0803","4-1_1","5"],

["0803","4-1_4","5"],

["0803","4-2_1","5"],

["0803","6-1_2","5"],

["0803","6-2_1","5"],

["0803","8-1_1","5"],

["0803","9-1_2","5"],

["0817","5-3_1","5"],

["0817","6-1_1","5"],

["0817","7-1_2","5"],

["0817","7-2_1","5"],

["0817","8-1_1","5"],

["0816","1-1_1","5"],

["0816","1-2_2","5"],

["0816","7-1_1","5"],

["0824_2","2-1_2","2.5"]]

motorname = np.array(motorname)

myad = []

syad = []

for mn in range(0,len(motorname)):

date = motorname[mn,0]

idx3 = motorname[mn,1]

fname = date+"\\m\\"+str(idx3)+".txt"

frame = 0

txt = open(fname, "r")

for i in range(0,40):

line = txt.readline().split('\t')

if (len(line) < 2):

break

frame = frame + 1

n = len(line)

txt.close()

motor = int(n/2)

for i in range(0,motor):

x = []

y = []

txt = open(fname, "r")

for j in range(0,frame):

line = txt.readline().split('\t')

x.append(line[i*2])

y.append(line[i*2+1])

globals()['x'+str(i)] = x

globals()['y'+str(i)] = y

txt.close()

for i in range(0,motor):

for j in range(0,frame):

if (len(globals()['x'+str(i)][j]) < 3):

globals()['x'+str(i)] = globals()['x'+str(i)][0:j]

globals()['y'+str(i)] = globals()['y'+str(i)][0:j]

break

for i in range(0,motor):

for j in range(0,len(globals()['x'+str(i)])):

globals()['x'+str(i)][j] = float(globals()['x'+str(i)][j])

globals()['y'+str(i)][j] = float(globals()['y'+str(i)][j])

for i in range(0,motor):

globals()['x'+str(i)] = np.array(globals()['x'+str(i)])

globals()['y'+str(i)] = np.array(globals()['y'+str(i)])

globals()['x'+str(i)] = globals()['x'+str(i)]*x100

globals()['y'+str(i)] = globals()['y'+str(i)]*x100

fname2 = date+"\\midline\\inert "+str(idx3)+" 1.txt"

n_pt = 0

txt2 = open(fname2, "r")

for i in range(0,3000):

line = txt2.readline().split('\t')

if (len(line) < 2):

break

n_pt = n_pt + 1

txt2.close()

midx = []

midy = []

txt2 = open(fname2, "r")

for i in range(0,n_pt):

line = txt2.readline().split('\t')

midx.append(line[0])

midy.append(line[1])

txt2.close()

mid0 = np.zeros((1,2))

mid0[0,0] = float(midx[0])

mid0[0,1] = float(midy[0])

mid0 = mid0/resize

mid0 = mid0*x100

#-------------------------------------------------------------------------------------------

for i in range(0,motor):

yad = []

counter = len(glob.glob1(date+"\\midline",str(idx3)+"*.txt"))

if(counter > len(globals()['x'+str(i)])):

nframe = len(globals()['x'+str(i)])

else:

nframe = counter

for j in range(0,nframe):

n_pt = 0

fname2 = date+"\\midline\\inert "+str(idx3)+" "+str(j+1)+".txt"

txt2 = open(fname2, "r")

for k in range(0,3000):

line = txt2.readline().split('\t')

if (len(line) < 2):

break

n_pt = n_pt + 1

txt2.close()

midx = []

midy = []

txt2 = open(fname2, "r")

for k in range(0,n_pt):

line = txt2.readline().split('\t')

midx.append(line[0])

midy.append(line[1])

txt2.close()

mid = np.zeros((n_pt,2))

mid[:,0] = np.array(midx)

mid[:,1] = np.array(midy)

wt_i = int(mid[n_pt-1,0])

wb_i = int(mid[n_pt-1,1])

mid = mid[0:n_pt-1,:]

n_pt = n_pt-1

mid = mid/resize

mid = mid*x100

num = 0

fname3 = date+"\\contour\\"+str(idx3)+" "+str(j+1)+".txt"

txt3 = open(fname3, "r")

for k in range(0,3000):

line = txt3.readline().split('\t')

if (len(line) < 2):

break

num = num + 1

txt3.close()

conx = []

cony = []

txt3 = open(fname3, "r")

for k in range(0,num):

line = txt3.readline().split('\t')

conx.append(line[0])

cony.append(line[1])

txt3.close()

con = np.zeros((num,2))

con[:,0] = np.array(conx)

con[:,1] = np.array(cony)

con = con/resize

con = con*x100

d1 = __d(mid0[0,:],mid[0,:])

d2 = __d(mid0[0,:],mid[n_pt-1,:])

if (d2 < d1):

mid = mid[::-1]

nwt_i = n_pt + 1- wb_i

nwb_i = n_pt + 1- wt_i

wt_i = nwt_i

wb_i = nwb_i

mid0[0,0] = mid[0,0]

mid0[0,1] = mid[0,1]

mid = __spline(mid,n_pt)

idx = __ind(globals()['x'+str(i)][j],globals()['y'+str(i)][j],mid)

m = int(n_pt/19)

if(m % 2 == 0):

m = m + 1

for w in range(0,3):

if (int(idx) - m < 0):

temp = np.array(mid[0:int(idx)+m,:])

idxm = int(len(temp)-m)

elif (int(idx) + m > n_pt):

temp = np.array(mid[int(idx)-m:n_pt,:])

idxm = m

else:

temp = np.array(mid[int(idx)-m:int(idx)+m,:])

idxm = m

popt, pcov = curve_fit(fitf, temp[:,0], temp[:,1])

m_l = np.array(temp)

m_l[:,0] = temp[:,0]

m_l[:,1] = popt[0]*temp[:,0]+popt[1]

jj = __ind(globals()['x'+str(i)][j],globals()['y'+str(i)][j],m_l)

idx = __ind(m_l[jj,0],m_l[jj,1],mid)

mtpd = 0

if(idx < int(n_pt/2)):

for w in range(0,idx-1):

mtpd = mtpd + __d(mid[w,:],mid[w+1,:])

if(idx >= int(n_pt/2)):

for w in range(idx,n_pt-1):

mtpd = mtpd + __d(mid[w,:],mid[w+1,:])

yad.append(mtpd)

if(yad[0] <= 0.62 and yad[0] > 0.0):

npf = float(motorname[i,2])

#ax1.plot(np.arange(0, len(yad)*npf, step=npf),yad,label = str(date)+" "+str(idx3))

ax1.plot(np.arange(0, len(yad)*npf, step=npf),yad,linewidth = 1.5,color = 'w')

popt, pcov = curve_fit(fitf, np.arange(0, len(yad)*npf, step=npf), yad)

myad.append(yad[0])

syad.append(popt[0])

if(yad[0] > 0.283):

ax2.plot(yad[0],popt[0],'o',markersize = 7,color = '#4393C3')

df.append(np.arange(0, len(yad)*npf, step=npf))

df.append(yad)

if(yad[0] <= 0.283):

ax2.plot(yad[0],popt[0],'o',markersize = 7,color = '#66C2A5')

df.append(np.arange(0, len(yad)*npf, step=npf))

df.append(yad)

print('--')

#-----------------------------------------------------------------

for ind in range(0,9):

if (ind == 0):

date = "0802"

nnn = 11

nn = [4,2,3,3,3,1,1,1,1,0,1]

mm = [2,5,3,1,3,3,3,4,3,2,1,4,3,3,2,1,1,1,5,5]

npf = 5

duration = 125

if (ind == 1):

date = "0815"

nnn = 4

nn = [2,2,4,3]

mm = [4,2,5,2,2,2,2,3,5,4,4]

npf = 5

if (ind == 2):

date = "0824"

nnn = 3

nn = [5,4,2]

mm = [2,3,1,1,3,1,2,3,3,1,1]

npf = 2.5

if (ind == 3):

date = "0725"

nnn = 10

nn = [4,3,4,6,4,4,2,2,2,3]

mm = [3,2,3,2,3,1,1,4,1,1,1,4,4,2,2,1,2,5,4,2,2,2,3,4,3,2,3,1,2,1,4,4,2,1]

npf = 5

if (ind == 4):

date = "0810"

nnn = 6

nn = [1,1,1,0,1,1]

mm = [2,1,2,2,4]

npf = 5

if (ind == 5):

date = "0803"

nnn = 11

nn = [0,2,2,4,4,2,3,2,2,2,1]

mm = [5,2,4,4,5,2,3,1,5,1,1,2,3,2,2,3,5,2,3,2,2,2,2,1]

npf = 5

if (ind == 6):

date = "0817"

nnn = 8

nn = [0,0,2,1,4,1,2,2]

mm = [2,2,1,1,2,2,1,1,2,1,2,1]

npf = 5

if (ind == 7):

date = "0816"

nnn = 9

nn = [2,1,0,1,1,2,2,1,2]

mm = [2,2,3,1,2,3,1,3,2,1,2,1]

npf = 5

if (ind == 8):

date = "0824_2"

nnn= 3

nn = [1,1,1]

mm = [3,2,1]

npf = 2.5

path = "D:\\desktop\\Tracking ver.5\\"+date

os.chdir(path)

mnum = -1

for t in range(0,nnn):

for u in range(0,int(nn[t])):

mnum = mnum + 1

for ww in range(0,int(mm[mnum])):

fname = "m\\"+str(t+1)+"-"+str(u+1)+"_"+str(ww+1)+".txt"

frame = 0

txt = open(fname, "r")

for i in range(0,40):

line = txt.readline().split('\t')

if (len(line) < 2):

break

frame = frame + 1

n = len(line)

txt.close()

motor = int(n/2)

for i in range(0,motor):

x = []

y = []

txt = open(fname, "r")

for j in range(0,frame):

line = txt.readline().split('\t')

x.append(line[i*2])

y.append(line[i*2+1])

globals()['x'+str(i)] = x

globals()['y'+str(i)] = y

txt.close()

for i in range(0,motor):

for j in range(0,frame):

if (len(globals()['x'+str(i)][j]) < 3):

globals()['x'+str(i)] = globals()['x'+str(i)][0:j]

globals()['y'+str(i)] = globals()['y'+str(i)][0:j]

break

for i in range(0,motor):

for j in range(0,len(globals()['x'+str(i)])):

globals()['x'+str(i)][j] = float(globals()['x'+str(i)][j])

globals()['y'+str(i)][j] = float(globals()['y'+str(i)][j])

for i in range(0,motor):

globals()['x'+str(i)] = np.array(globals()['x'+str(i)])

globals()['y'+str(i)] = np.array(globals()['y'+str(i)])

globals()['x'+str(i)] = globals()['x'+str(i)]*x100

globals()['y'+str(i)] = globals()['y'+str(i)]*x100

fname2 = "midline\\"+str(t+1)+"-"+str(u+1)+"_"+str(ww+1)+" 1.txt"

n_pt = 0

txt2 = open(fname2, "r")

for i in range(0,3000):

line = txt2.readline().split('\t')

if (len(line) < 2):

break

n_pt = n_pt + 1

txt2.close()

midx = []

midy = []

txt2 = open(fname2, "r")

for i in range(0,n_pt):

line = txt2.readline().split('\t')

midx.append(line[0])

midy.append(line[1])

txt2.close()

mid0 = np.zeros((1,2))

mid0[0,0] = float(midx[0])

mid0[0,1] = float(midy[0])

mid0 = mid0/resize

mid0 = mid0*x100

fnamew = "division\\"+str(t+1)+"-"+str(u+1)+"_"+str(ww+1)+".txt"

ndiv = 0

txt = open(fnamew, "r")

for h in range(0,1000):

line = txt.readline().split('\n')

if (len(line) < 2):

break

ndiv = ndiv + 1

txt.close()

divx = []

txt = open(fnamew, "r")

for h in range(0,ndiv):

line = txt.readline().split('\t')

divx.append(line[0])

txt.close()

div = np.array(divx,dtype = "int")

#-------------------------------------------------------------------------------------------

for i in range(0,motor):

yad = []

counter = len(glob.glob1("midline",str(t+1)+"-"+str(u+1)+"_"+str(ww+1)+"*.txt"))

if(counter > len(globals()['x'+str(i)])):

nframe = len(globals()['x'+str(i)])

else:

nframe = counter

if(len(div) > 0):

if(div[0] < nframe):

nframe = div[0]

for j in range(0,nframe):

n_pt = 0

fname2 = "midline\\"+str(t+1)+"-"+str(u+1)+"_"+str(ww+1)+" "+str(j+1)+".txt"

txt2 = open(fname2, "r")

for k in range(0,3000):

line = txt2.readline().split('\t')

if (len(line) < 2):

break

n_pt = n_pt + 1

txt2.close()

midx = []

midy = []

txt2 = open(fname2, "r")

for k in range(0,n_pt):

line = txt2.readline().split('\t')

midx.append(line[0])

midy.append(line[1])

txt2.close()

mid = np.zeros((n_pt,2))

mid[:,0] = np.array(midx)

mid[:,1] = np.array(midy)

wt_i = int(mid[n_pt-1,0])

wb_i = int(mid[n_pt-1,1])

mid = mid[0:n_pt-1,:]

n_pt = n_pt-1

mid = mid/resize

mid = mid*x100

num = 0

fname3 = "contour\\"+str(t+1)+"-"+str(u+1)+"_"+str(ww+1)+" "+str(j+1)+".txt"

txt3 = open(fname3, "r")

for k in range(0,3000):

line = txt3.readline().split('\t')

if (len(line) < 2):

break

num = num + 1

txt3.close()

conx = []

cony = []

txt3 = open(fname3, "r")

for k in range(0,num):

line = txt3.readline().split('\t')

conx.append(line[0])

cony.append(line[1])

txt3.close()

con = np.zeros((num,2))

con[:,0] = np.array(conx)

con[:,1] = np.array(cony)

con = con/resize

con = con*x100

d1 = __d(mid0[0,:],mid[0,:])

d2 = __d(mid0[0,:],mid[n_pt-1,:])

if (d2 < d1):

mid = mid[::-1]

nwt_i = n_pt + 1- wb_i

nwb_i = n_pt + 1- wt_i

wt_i = nwt_i

wb_i = nwb_i

mid0[0,0] = mid[0,0]

mid0[0,1] = mid[0,1]

mid = __spline(mid,n_pt)

idx = __ind(globals()['x'+str(i)][j],globals()['y'+str(i)][j],mid)

m = int(n_pt/19)

if(m % 2 == 0):

m = m + 1

for w in range(0,3):

if (int(idx) - m < 0):

temp = np.array(mid[0:int(idx)+m,:])

idxm = int(len(temp)-m)

elif (int(idx) + m > n_pt):

temp = np.array(mid[int(idx)-m:n_pt,:])

idxm = m

else:

temp = np.array(mid[int(idx)-m:int(idx)+m,:])

idxm = m

popt, pcov = curve_fit(fitf, temp[:,0], temp[:,1])

m_l = np.array(temp)

m_l[:,0] = temp[:,0]

m_l[:,1] = popt[0]*temp[:,0]+popt[1]

jj = __ind(globals()['x'+str(i)][j],globals()['y'+str(i)][j],m_l)

idx = __ind(m_l[jj,0],m_l[jj,1],mid)

mtpd = 0

if(idx < int(n_pt/2)):

for w in range(0,idx-1):

mtpd = mtpd + __d(mid[w,:],mid[w+1,:])

if(idx >= int(n_pt/2)):

for w in range(idx,n_pt-1):

mtpd = mtpd + __d(mid[w,:],mid[w+1,:])

yad.append(mtpd)

if(yad[0] <= 1.3 and yad[0] > 0.62):

if(len(yad) > 5):

popt, pcov = curve_fit(fitf, np.arange(0, len(yad)*npf, step=npf), yad)

if(yad[len(yad)-1] < 2.25):

ax1.plot(np.arange(0, len(yad)*npf, step=npf),yad,linewidth = 1.5,color = 'w')

if(yad[len(yad)-1] > 2.25):

ax1.plot(np.arange(0, len(yad)*npf, step=npf),yad,linewidth = 0.5,color = 'w')

if(popt[0] > 0.0009):

ax2.plot(yad[0],popt[0],'o',markersize = 7,color = '#4393C3')

df.append(np.arange(0, len(yad)*npf, step=npf))

df.append(yad)

myad.append(yad[0])

syad.append(popt[0])

#-----------------------------------------------------------------

print('--')

for i in range(0,2):

if (i == 0):

date = "0716_2"

nnn = 10

nn = [2,0,1,1,1,2,3,1,0,1]

mm = [1,1,1,1,1,1,1,1,1,1,1,1]

if(i == 1):

date = "0716"

nnn = 10

nn = [0,4,0,1,2,3,3,1,0,2]

mm = [2,1,1,1,1,1,1,1,1,1,1,1,2,1,1,1]

path = "D:\\desktop\\Tracking ver.5\\"+date

os.chdir(path)

mnum = -1

npf = 5

for t in range(0,nnn):

for u in range(0,int(nn[t])):

mnum = mnum + 1

for ww in range(0,int(mm[mnum])):

fname = "py\\"+str(t+1)+"-"+str(u+1)+"_"+str(ww+1)+".txt"

txt = open(fname, "r")

py = []

for i in range(0,30):

line = txt.readline().split('\n')

if(line[0] == ''):

break

py.append(float(line[0]))

txt.close()

py = np.array(py)

popt, pcov = curve_fit(fitf, np.arange(0, len(py)*npf, step=npf), py)

if(popt[0] < -0.0009):

continue

myad.append(py[0])

syad.append(popt[0])

ax1.plot(np.arange(0, len(py)*npf, step=npf),py)

if(py[0] <= 0.283):

ax2.plot(py[0],popt[0],'o',markersize = 7, color = '#66C2A5')

df.append(np.arange(0, len(py)*npf, step=npf))

df.append(py)

if(py[0] > 0.283):

ax2.plot(py[0],popt[0],'o',markersize = 7, color = '#4393C3')

df.append(np.arange(0, len(py)*npf, step=npf))

df.append(py)

#__________________________________________________________________________________

myad = np.array(myad)

syad = np.array(syad)

p , e = curve_fit(piecewise_linear, myad,syad)

print("x0:"+str(p[0]), "k2:"+str(p[1]))

#ax2.plot(myad,syad,'o')

#ax1.legend()

#ax2.plot([0,0],[1.6,0],'--')

#ax2.plot([p[0],p[0]],[-0.005,0.09],'b--')

fs = 20

ax1.set_xlabel('Time (min)',fontsize = fs)

ax1.set_ylabel('Axial position ($\mathregular{P_y}$)\nfrom the nearest pole (µm)',fontsize = fs)

ax2.set_ylabel('Axial velocity ($\mathregular{V_{P_y}}$)(µm/min)',fontsize = fs)

ax2.set_xlabel('Axial position ($\mathregular{P_y}$)\nfrom the nearest pole (µm)',fontsize = fs)

ax1.set_yticks(np.arange(0,240, step=60))

ax1.set_yticks(np.arange(0,3, step=1))

ax1.axis([0,190,0,2.25])

ax2.set_yticks(np.arange(0,0.06, step=0.02))

ax2.set_xticks(np.arange(0,3, step=1))

ax2.axis([-0.05,1.3,-0.001,0.025])

ax2.plot(np.arange(0,2.21, step=0.01),piecewise_linear(np.arange(0,2.21, step=0.01), *p),color = 'k',linewidth = 1.5)

#ax2.xticks(np.arange(-0.5,0.6, step=0.1))

#ax2.yticks(np.arange(-0.5,0.6, step=0.1))

ax1.tick_params(direction='in', length=6, width=1.5)

ax2.tick_params(direction='in', length=6, width=1.5)

plt.tight_layout()

plt.show()

#plt.savefig("C:\\Users\\elain\\Desktop\\figures\\1\\1bc.svg")

dff = DataFrame(df)

dff.to_csv('C:\\Users\\elain\\Desktop\\data example\\test.csv')

- Relative axial velocity vs relative axial distance (Figure 2C)

import numpy as np

import scipy as sp

import matplotlib.pyplot as plt

import os

from scipy.optimize import curve_fit

import matplotlib as mpl

import matplotlib.ticker as mticker

from cycler import cycler

mpl.rcParams['axes.linewidth'] = 1.5

label_size = 15

mpl.rcParams['xtick.labelsize'] = label_size

mpl.rcParams['ytick.labelsize'] = label_size

mpl.rcParams["font.family"] = 'serif'

# Get current size

fig_size = plt.rcParams["figure.figsize"]

# Prints: [8.0, 6.0]

print("Current size:", fig_size)

# Set figure width to 12 and height to 9

fig_size[0] = 11.2

fig_size[1] = 6

plt.rcParams["figure.figsize"] = fig_size

plt.rcParams['mathtext.fontset'] = 'dejavuserif'

path = "C:\\Users\\elain\\Desktop\\Tracking ver.5\\hubble"

os.chdir(path)

def f(x, a, b): # this is your 'straight line' y=f(x)

return a*x + b

t = []

for i in range(0,15):

fnamer = str(i+1)+".txt"

txt = open(fnamer, "r")

yad = []

v = []

if(i+1 < 6):

c = '#35978F'

elif(i+1 > 10):

c = '#4393C3'

else:

c = '#D6604D'

for j in range(0,60):

line = txt.readline().split('\t')

if (line[0] == ''):

txt.close()

break

yad.append(float(line[0]))

v.append(float(line[1]))

if(j == 0):

t.append(int(line[2]))

yad = np.array(yad)

v = np.array(v)

popt, pcov = curve_fit(f,yad,v)

print(t[i], popt[0])

residuals = v - f(yad, popt[0],popt[1])

ss_res = np.sum(residuals**2)

ss_tot = np.sum((v-np.mean(v))**2)

r2 = 1 - (ss_res / ss_tot)

ff = mticker.ScalarFormatter(useOffset=False, useMathText=True)

g = lambda x,pos : "${}$".format(ff._formatSciNotation('%1.10e' % x))

fmt = mticker.FuncFormatter(g)

plt.subplot(3,5,i+1)

ax = plt.gca()

ax.tick_params(direction='in', length=6, width=1.5)

plt.plot([0,0],[-0.008,0.07],'k',linewidth = 0.7,color = '#C8C8C8')

plt.plot([-0.5,5],[0,0],'k',linewidth = 0.7,color = '#C8C8C8')

plt.plot(yad,v,'o',markersize = 4,color = c)

plt.plot(yad,yad*popt[0]+popt[1],linewidth = 1.5,color = c)

plt.yticks(np.arange(0,0.12,0.03))

plt.xticks(np.arange(0,5,2))

plt.text(0.4,0.05,'$\mathregular{t}$ = '+str(t[i]),fontsize = 15)

plt.axis([-0.5,5,-0.008,0.07])

#plt.xlabel("Relative lateral distance ($\mathregular{N_y}$)\n(µm)",fontsize = 15)

#plt.ylabel("Relative lateral velocity ($\mathregular{V_{D_x}}$)\n (µm/min)",fontsize = 15)

plt.tight_layout()

plt.savefig("C:\\Users\\elain\\Desktop\\3x5.svg")

#plt.show()

- Axial growth rate vs time (Figure 2D)

import numpy as np

import scipy as sp

import matplotlib.pyplot as plt

import os

from scipy.optimize import curve_fit

import matplotlib as mpl

import matplotlib.ticker as mticker

from cycler import cycler

mpl.rcParams['axes.linewidth'] = 1.5

label_size = 20

mpl.rcParams['xtick.labelsize'] = label_size

mpl.rcParams['ytick.labelsize'] = label_size

mpl.rcParams["font.family"] = 'serif'

# Get current size

fig_size = plt.rcParams["figure.figsize"]

# Prints: [8.0, 6.0]

print("Current size:", fig_size)

# Set figure width to 12 and height to 9

fig_size[0] = 6

fig_size[1] = 5.5

plt.rcParams["figure.figsize"] = fig_size

plt.rcParams['mathtext.fontset'] = 'dejavuserif'

path = "D:\\desktop\\Tracking ver.5\\"

os.chdir(path)

def f(x, a, b): # this is your 'straight line' y=f(x)

return a*x + b

'''

#cm = ['#D6604D','#4393C3','#B31B2D','#35978F']

cm = ['#2166AC']

mpl.rcParams['axes.prop_cycle'] = cycler('color', cm)

'''

n = 0

ax = plt.gca()

ax.tick_params(direction='in', length=6, width=1.5)

for i in range(0,11):

print('~'+str(i+1)+'~')

fnamer = "hubble shadow\\"+str(i+1)+".txt"

txt = open(fnamer, "r")

t = []

H = []

for j in range(0,10):

line = txt.readline().split('\t')

if (line[0] == ''):

txt.close()

break

line = np.array(line)

t.append(int(line[0])-20)

H.append(line[1])

if(i == 4):

plt.plot(t,H,'o-',color = '#35978F')

elif(i ==5):

plt.plot(t,H,'o-',color = '#D6604D')

elif(i ==6):

plt.plot(t,H,'o-',color = '#4393C3')

else:

plt.plot(t,H,'o-',color = 'gray', markerfacecolor='none')

ax = plt.gca()

ax.tick_params(direction='in', length=6, width=1.5)

plt.xlim(0,40)

plt.ylim(0,0.025)

plt.xlabel("Time (min)",fontsize = 22)

plt.ylabel("Growth rate (1/min)",fontsize = 22)

plt.tight_layout()

plt.show()

- Normalized relative axial velocity vs relative axial distance (Supplymentary Fig. 4A)

import numpy as np

import scipy as sp

import matplotlib.pyplot as plt

import os

from scipy.optimize import curve_fit

import matplotlib as mpl

import matplotlib.ticker as mticker

from cycler import cycler

mpl.rcParams['axes.linewidth'] = 1.5

label_size = 20

mpl.rcParams['xtick.labelsize'] = label_size

mpl.rcParams['ytick.labelsize'] = label_size

mpl.rcParams["font.family"] = 'serif'

# Get current size

fig_size = plt.rcParams["figure.figsize"]

# Prints: [8.0, 6.0]

print("Current size:", fig_size)

# Set figure width to 12 and height to 9

fig_size[0] = 6

fig_size[1] = 5.5

plt.rcParams["figure.figsize"] = fig_size

plt.rcParams['mathtext.fontset'] = 'dejavuserif'

path = "D:\\desktop\\Tracking ver.5\\"

os.chdir(path)

def f(x, a, b): # this is your 'straight line' y=f(x)

return a*x + b

#cm = ['#D6604D','#4393C3','#B31B2D','#35978F']

cm = ['#2166AC']

mpl.rcParams['axes.prop_cycle'] = cycler('color', cm)

n = 0

ax = plt.gca()

ax.tick_params(direction='in', length=6, width=1.5)

ymax = 0

vmax = 0

for i in range(0,14):

print('~'+str(i+1)+'~')

fnamer = "hubble norm\\"+str(i+1)+".txt"

txt = open(fnamer, "r")

for j in range(0,10):

line = txt.readline().split('\t')

if (line[0] == ''):

txt.close()

break

line = np.array(line)

ind = np.where(line == '')

ind0 = ind[0][0]-1

y = line[1:1+ind0]

y = np.array(y,dtype = 'float')

v = line[ind0+2:ind0+2+ind0]

v = np.array(v,dtype = 'float')

popt, pcov = curve_fit(f,y,v)

plt.plot(y,v/popt[0],'o', markersize = 5)

for k in range(0,len(y)):

print(y[k],v[k]/popt[0])

print(' ')

n = n + 1

plt.xlabel("Relative axial distance ($\mathregular{V_{max}}$)\n(µm)",fontsize = 22)

plt.ylabel("Relative axial Normalized velocity ($\mathregular{V_{D_y}}$)\n (µm/min)",fontsize = 22)

plt.xlim(-0.3,4.1)

plt.ylim(-0.3,4.1)

print(n)

plt.tight_layout()

plt.show()

- Relative lateral velocity vs relative lateral distance (Figure 2F)

import numpy as np

import scipy as sp

import matplotlib.pyplot as plt

import os

from scipy.optimize import curve_fit

import matplotlib as mpl

import matplotlib.ticker as mticker

from cycler import cycler

mpl.rcParams['axes.linewidth'] = 1.5

label_size = 15

mpl.rcParams['xtick.labelsize'] = label_size

mpl.rcParams['ytick.labelsize'] = label_size

mpl.rcParams["font.family"] = 'serif'

# Get current size

fig_size = plt.rcParams["figure.figsize"]

# Prints: [8.0, 6.0]

print("Current size:", fig_size)

# Set figure width to 12 and height to 9

fig_size[0] = 11.2

fig_size[1] = 6

plt.rcParams["figure.figsize"] = fig_size

plt.rcParams['mathtext.fontset'] = 'dejavuserif'

path = "C:\\Users\\elain\\Desktop\\Tracking ver.5\\x hubble"

os.chdir(path)

def f(x, a, b): # this is your 'straight line' y=f(x)

return a*x + b

t = []

for i in range(0,15):

fnamer = str(i+1)+".txt"

txt = open(fnamer, "r")

yad = []

v = []

if(i+1 < 6):

c = '#35978F'

elif(i+1 > 10):

c = '#4393C3'

else:

c = '#D6604D'

for j in range(0,60):

line = txt.readline().split('\t')

if (line[0] == ''):

txt.close()

break

yad.append(float(line[0]))

v.append(float(line[1]))

if(j == 0):

t.append(int(line[2]))

yad = np.array(yad)

v = np.array(v)

popt, pcov = curve_fit(f,yad,v)

print(t[i], popt[0])

residuals = v - f(yad, popt[0],popt[1])

ss_res = np.sum(residuals**2)

ss_tot = np.sum((v-np.mean(v))**2)

r2 = 1 - (ss_res / ss_tot)

ff = mticker.ScalarFormatter(useOffset=False, useMathText=True)

g = lambda x,pos : "${}$".format(ff._formatSciNotation('%1.10e' % x))

fmt = mticker.FuncFormatter(g)

plt.subplot(3,5,i+1)

ax = plt.gca()

ax.tick_params(direction='in', length=6, width=1.5)

#plt.plot([0,0],[-0.008,0.07],'k',linewidth = 0.7,color = '#C8C8C8')

plt.plot([-0.5,5],[0,0],'k',linewidth = 0.7,color = '#C8C8C8')

plt.plot(yad,v,'o',markersize = 4,color = c)

plt.plot(yad,yad*popt[0]+popt[1],linewidth = 1.5,color = c)

plt.yticks(np.arange(0,0.04,0.02))

#plt.xticks(np.arange(0,0.6,0.3))

#plt.text(0.4,0.05,'$\mathregular{t}$ = '+str(t[i]),fontsize = 15)

plt.axis([0,1.2,-0.03,0.03])

#plt.xlabel("Relative lateral distance ($\mathregular{N_y}$)\n(µm)",fontsize = 15)

#plt.ylabel("Relative lateral velocity ($\mathregular{V_{D_x}}$)\n (µm/min)",fontsize = 15)

plt.tight_layout()

plt.savefig("C:\\Users\\elain\\Desktop\\x 3x5.svg")

#plt.show()

- Lateral growth rate vs time (Figure 2G)

import numpy as np

import scipy as sp

import matplotlib.pyplot as plt

import os

from scipy.optimize import curve_fit

import matplotlib as mpl

import matplotlib.ticker as mticker

from cycler import cycler

mpl.rcParams['axes.linewidth'] = 1.5

label_size = 20

mpl.rcParams['xtick.labelsize'] = label_size

mpl.rcParams['ytick.labelsize'] = label_size

mpl.rcParams["font.family"] = 'serif'

# Get current size

fig_size = plt.rcParams["figure.figsize"]

# Prints: [8.0, 6.0]

print("Current size:", fig_size)

# Set figure width to 12 and height to 9

fig_size[0] = 6

fig_size[1] = 5.5

plt.rcParams["figure.figsize"] = fig_size

plt.rcParams['mathtext.fontset'] = 'dejavuserif'

path = "D:\\desktop\\Tracking ver.5\\"

os.chdir(path)

def f(x, a, b): # this is your 'straight line' y=f(x)

return a*x + b

'''

#cm = ['#D6604D','#4393C3','#B31B2D','#35978F']

cm = ['#2166AC']

mpl.rcParams['axes.prop_cycle'] = cycler('color', cm)

'''

n = 0

ax = plt.gca()

ax.tick_params(direction='in', length=6, width=1.5)

for i in range(0,7):

print('~'+str(i+1)+'~')

fnamer = "x hubble shadow\\"+str(i+1)+".txt"

txt = open(fnamer, "r")

t = []

H = []

for j in range(0,10):

line = txt.readline().split('\t')

if (line[0] == ''):

txt.close()

break

line = np.array(line)

t.append(int(line[0])-20)

H.append(line[1])

if(i == 4):

plt.plot(t,H,'o-',color = '#35978F')

elif(i ==5):

plt.plot(t,H,'o-',color = '#D6604D')

elif(i ==6):

plt.plot(t,H,'o-',color = '#4393C3')

else:

plt.plot(t,H,'o-',color = '#898989', markerfacecolor='none')

#plt.plot(t,H,'o-')

ax = plt.gca()

ax.tick_params(direction='in', length=6, width=1.5)

plt.xlim(0,40)

plt.ylim(-0.03,0.03)

plt.xlabel("Time (min)",fontsize = 22)

plt.ylabel("Growth rate (1/min)",fontsize = 22)

plt.tight_layout()

plt.show()

- Relative lateral velocity vs relative lateral distance (Figure 2H)

import numpy as np

import scipy as sp

import matplotlib.pyplot as plt

import os

from scipy.optimize import curve_fit

import matplotlib as mpl

import matplotlib.ticker as mticker

from cycler import cycler

import pandas as pd

from pandas.core.frame import DataFrame

mpl.rcParams['axes.linewidth'] = 1.5

label_size = 20

mpl.rcParams['xtick.labelsize'] = label_size

mpl.rcParams['ytick.labelsize'] = label_size

mpl.rcParams["font.family"] = 'serif'

# Get current size

fig_size = plt.rcParams["figure.figsize"]

# Prints: [8.0, 6.0]

print("Current size:", fig_size)

# Set figure width to 12 and height to 9

fig_size[0] = 6

fig_size[1] = 5.5

plt.rcParams["figure.figsize"] = fig_size

plt.rcParams['mathtext.fontset'] = 'dejavuserif'

path = "D:\\desktop\\Tracking ver.5\\"

os.chdir(path)

def f(x, a, b): # this is your 'straight line' y=f(x)

return a*x + b

#cm = ['#D6604D','#4393C3','#B31B2D','#35978F']

cm = ['#2166AC']

mpl.rcParams['axes.prop_cycle'] = cycler('color', cm)

n = 0

ax = plt.gca()

ax.tick_params(direction='in', length=6, width=1.5)

ymax = 0

vmax = 0

df = []

for i in range(0,14):

#print('~'+str(i+1)+'~')

fnamer = "x hubble norm\\"+str(i+1)+".txt"

txt = open(fnamer, "r")

for j in range(0,10):

line = txt.readline().split('\t')

if (line[0] == ''):

txt.close()

break

line = np.array(line)

ind = np.where(line == '')

ind0 = ind[0][0]-1

y = line[1:1+ind0]

y = np.array(y,dtype = 'float')

v = line[ind0+2:ind0+2+ind0]

v = np.array(v,dtype = 'float')

plt.plot(y,v,'o',markersize = 5)

df.append(y)

df.append(v)

n = n + 1

for w in range(0,len(v)):

print(v[w])

dff = DataFrame(df)

dff.to_csv('C:\\Users\\elain\\Desktop\\data example\\test.csv')

plt.xlabel("Relative axial distance ($\mathregular{D_x}$)\n(µm)",fontsize = 22)

plt.ylabel("Relative axial velocity ($\mathregular{V_{D_x}}$)\n (µm/min)",fontsize = 22)

plt.xlim(0,1.2)

plt.ylim(-0.03,0.03)

print(n)

plt.tight_layout()

plt.show()

- Tracing the movement of BFMs in the normalized coordinate (Figure 3BCED)

import numpy as np

import scipy as sp

import matplotlib.pyplot as plt

import os

from scipy.optimize import curve_fit

import matplotlib as mpl

from cycler import cycler

import pandas as pd

from pandas.core.frame import DataFrame

def f(x, a, b): # this is your 'straight line' y=f(x)

return a*x + b

fig_size = plt.rcParams["figure.figsize"]

fig_size[0] = 9

fig_size[1] = 7.2

plt.rcParams["figure.figsize"] = fig_size

mpl.rcParams['axes.linewidth'] = 1.5

label_size = 12

mpl.rcParams['xtick.labelsize'] = label_size

mpl.rcParams['ytick.labelsize'] = label_size

mpl.rcParams["font.family"] = 'serif'

mpl.rcParams['axes.prop_cycle'] = cycler('color', ['#D6604D'

,'#B31B2D'

,'#4393C3'

,'#2166AC'

,'#66C2A5'

,'#35978F'])

df = []

#-----------------------------------------------------------

fig = plt.figure(0)

ax1=fig.add_subplot(2,2,1)

ax2=fig.add_subplot(2,2,3)

ax3=fig.add_subplot(2,2,2)

ax4=fig.add_subplot(2,2,4)

ax1.tick_params(direction='in', length=6, width=1.5)

ax2.tick_params(direction='in', length=6, width=1.5)

ax3.tick_params(direction='in', length=6, width=1.5)

ax4.tick_params(direction='in', length=6, width=1.5)

path = "D:\\desktop\\Tracking ver.5\\"

os.chdir(path)

divitime = []

n = 0

fname = "contract2.txt"

txt = open(fname, "r")

for i in range(0,300):

line = txt.readline().split('\t')

if(len(line) < 4):

txt.close()

break

cp = int(line[2])

npf = float(line[3])

fyrd = line[0]+'\\y rd\\'+line[1]+'.txt'

yrd = []

txt2 = open(fyrd, "r")

for j in range(0,50):

line2 = txt2.readline().split('\t')

if(line2[0] == ''):

txt2.close()

break

yrd.append(float(line2[0]))

fdiv = line[0]+'\\division\\'+line[1]+'.txt'

div = []

txt3 = open(fdiv, "r")

for j in range(0,10):

line3 = txt3.readline().split('\t')

if(line3[0] == ''):

txt3.close()

break

div.append(int(line3[0]))

if(cp >= 5 and div[0]-cp >= 2):

el = yrd[0:cp-2]

divi = yrd[cp-3:div[0]]

#print(len(el)*npf,len(divi)*npf)

if(len(divi) < 10):

n = n + 1

ax1.plot(np.arange(0,len(el)*npf, step=npf),el,linewidth = 1.8)

ax2.plot(np.arange(0,len(divi)*npf, step=npf),divi,linewidth = 1.8)

popte, pcove = curve_fit(f, np.arange(0,len(el)*npf, step=npf), el)

ax3.plot(popte[0],el[0],'o')

poptd, pcovd = curve_fit(f, np.arange(0,len(divi)*npf, step=npf), divi)

#print(round(popte[0],5),round(el[0],5))

if(divi[0] > 0):

ax4.plot(poptd[0],divi[0],'o')

print(round(poptd[0],5),round(divi[0],5))

if(divi[0] < 0):

ax4.plot(poptd[0]*-1,divi[0],'o')

print(round(poptd[0],5),round(divi[0],5))

fs = 15

ax1.spines['bottom'].set_position('center')

ax1.spines['right'].set_color('none')

ax1.spines['top'].set_color('none')

ax2.spines['bottom'].set_position('center')

ax2.spines['right'].set_color('none')

ax2.spines['top'].set_color('none')

ax3.spines['bottom'].set_position('center')

ax3.spines['left'].set_position('center')

ax3.spines['right'].set_color('none')

ax3.spines['top'].set_color('none')

ax4.spines['bottom'].set_position('center')

ax4.spines['left'].set_position('center')

ax4.spines['right'].set_color('none')

ax4.spines['top'].set_color('none')

ax2.set_xlabel('Time (min)', fontsize=fs)

ax2.set_ylabel('$\mathregular{N_y}$', fontsize=fs)

ax1.xaxis.set_label_coords(0.85, 0.45)

ax2.xaxis.set_label_coords(0.85, 0.45)

ax3.xaxis.set_label_coords(0.85, 0.45)

ax4.xaxis.set_label_coords(0.85, 0.45)

ax2.set_xticks(np.arange(0,100, step=20))

ax1.set_xticks(np.arange(0,100, step=20))

ax1.axis([0,80,-0.5,0.5])

ax2.axis([0,80,-0.5,0.5])

ax3.axis([-0.005,0.005,-0.5,0.5])

ax4.axis([-0.005,0.005,-0.5,0.5])

ax1.set_yticks(np.arange(-0.5,0.6, step=0.2))

ax3.set_yticks(np.arange(-0.5,0.6, step=0.2))

ax4.set_yticks(np.arange(-0.5,0.6, step=0.2))

#ax3.set_xticks(np.arange(-0.01,0.015, step=0.005))

#ax4.set_xticks(np.arange(-0.01,0.015, step=0.005))

ax1.set_xlabel('Time (min)', fontsize=fs)

ax1.set_ylabel('Normalized axial position ($\mathregular{N_y}$)', fontsize=fs)

ax2.set_yticks(np.arange(-0.5,0.6, step=0.2))

ax3.set_xlabel('Slope (1/min)', fontsize=fs)

ax4.set_xlabel('Slope (1/min)', fontsize=fs)

print(n)

dff = DataFrame(df)

dff.to_csv('C:\\Users\\elain\\Desktop\\data example\\test.csv')

plt.tight_layout()

plt.show()

#plt.savefig("C:\\Users\\elain\\Desktop\\figures\\2&3\\3ab.svg")

- Tracking of BFMs’ positions across two generations (Figure 4C)

import numpy as np

import scipy as sp

import matplotlib.pyplot as plt

import os

from scipy.optimize import curve_fit

import matplotlib as mpl

mpl.rcParams['axes.linewidth'] = 1.5

label_size = 25

mpl.rcParams['xtick.labelsize'] = label_size

mpl.rcParams['ytick.labelsize'] = label_size

# Get current size

fig_size = plt.rcParams["figure.figsize"]

# Set figure width to 12 and height to 9

fig_size[0] = 7

fig_size[1] = 6.35

plt.rcParams["figure.figsize"] = fig_size

mpl.rcParams["font.family"] = 'serif'

def f(x, a, b): # this is your 'straight line' y=f(x)

return a*x + b

ax = plt.gca()

ax.tick_params(direction='in', length=6, width=1.5)

#-----------------------------------------------------

n = 0

upperb = 0.5

lowb = -0.5

xp = []

xn = []

yp = []

yn = []

for ind in range(0,9):

if (ind == 0):

date = "0802"

nnn = 11

nn = [4,2,3,3,3,1,1,1,1,0,1]

mm = [2,5,3,1,3,3,3,4,3,2,1,4,3,3,2,1,1,1,5,5]

npf = 5

duration = 125

if (ind == 1):

date = "0815"

nnn = 4

nn = [2,2,4,3]

mm = [4,2,5,2,2,2,2,3,5,4,4]

npf = 5

if (ind == 2):

date = "0824"

nnn = 3

nn = [5,4,2]

mm = [2,3,1,1,3,1,2,3,3,1,1]

npf = 2.5

if (ind == 3):

date = "0725"

nnn = 10

nn = [4,3,4,6,4,4,2,2,2,3]

mm = [3,2,3,2,3,1,1,4,1,1,1,4,4,2,2,1,2,5,4,2,2,2,3,4,3,2,3,1,2,1,4,4,2,1]

npf = 5

if (ind == 4):

date = "0810"

nnn = 6

nn = [1,1,1,0,1,1]

mm = [2,1,2,2,4]

npf = 5

if (ind == 5):

date = "0803"

nnn = 11

nn = [0,2,2,4,4,2,3,2,2,2,1]

mm = [5,2,4,4,5,2,3,1,5,1,1,2,3,2,2,3,5,2,3,2,2,2,2,1]

npf = 5

if (ind == 6):

date = "0817"

nnn = 8

nn = [0,0,2,1,4,1,2,2]

mm = [2,2,1,1,2,2,1,1,2,1,2,1]

npf = 5

if (ind == 7):

date = "0816"

nnn = 9

nn = [2,1,0,1,1,2,2,1,2]

mm = [2,2,3,1,2,3,1,3,2,1,2,1]

npf = 5

if (ind == 8):

date = "0824_2"

nnn= 3

nn = [1,1,1]

mm = [3,2,1]

npf = 2.5

path = "D:\\desktop\\Tracking ver.5\\"+date

os.chdir(path)

mnum = -1

for t in range(0,nnn):

for u in range(0,int(nn[t])):

mnum = mnum + 1

for ww in range(0,int(mm[mnum])):

fnamew = "y rd\\"+str(t+1)+"-"+str(u+1)+"_"+str(ww+1)+".txt"

txt = open(fnamew, "r")

yrd = []

for i in range(0,60):

line = txt.readline().split('\n')

if (line[0] == ''):

txt.close()

break

yrd.append(float(line[0]))

fnamew = "division\\"+str(t+1)+"-"+str(u+1)+"_"+str(ww+1)+".txt"

div = [0]

txt2 = open(fnamew, "r")

for i in range(0,10):

line2 = txt2.readline().split('\t')

if(line2[0] == ''):

txt2.close()

break

div.append(int(line2[0]))

#print(len(div))

if(len(yrd) > 0 and len(div) <= 4):

xpp = []

ypp = []

for i in range(0,len(div)-1):

if(len(yrd) > div[i+1] ):

n = n + 1

x0 = yrd[div[i]]

x1 = yrd[div[i+1]]

print(x0,x1)

if(x1 > x0*2 ):

xp.append(x0)

yp.append(x1)

xpp.append(x0)

ypp.append(x1)

if(x1 < x0*2):

xn.append(x0)

yn.append(x1)

xpp.append(x0)

ypp.append(x1)

plt.plot(xpp,ypp,'o',markevery = 3)

print(n)

plt.plot([0,0.5],[-0.5,0.5],color = 'k',linewidth = 1.5)

plt.plot([-0.5,0],[-0.5,0.5],color = 'k',linewidth = 1.5)

plt.plot([-0.5,0.5],[-0.5,0.5],color = 'k',linestyle = '--',linewidth = 1.5)

plt.ylabel("$\mathregular{N_{y(n+1)}}$",fontsize = 25)

plt.xlabel("$\mathregular{N_{y(n)}}$",fontsize = 25)

plt.axis([-0.5,0.5,-0.5,0.5])

plt.xticks(np.arange(-0.3,0.6, step=0.2))

plt.yticks(np.arange(-0.5,0.6, step=0.2))

#plt.plot(xp,yp,'o',color = '#4393C3')

#plt.plot(xn,yn,'o',color = '#4393C3')

poptp, pcovp = curve_fit(f,xp,yp)

poptn, pcovn = curve_fit(f,xn,yn)

plt.tight_layout()

plt.show()

#plt.savefig("C:\\Users\\elain\\Desktop\\figures\\4\\4b.svg")

- BFMs axial distribution in the normalized coordinate (Figure 4F)

import numpy as np

import scipy as sp

import os

import matplotlib.pyplot as plt

import matplotlib as mpl

plt.rcParams["figure.figsize"] = [7,5.7]

mpl.rcParams['axes.linewidth'] = 1.5

mpl.rcParams['font.family'] = 'serif'

plt.rcParams['xtick.labelsize']=20

plt.rcParams['ytick.labelsize']=20

fig = plt.figure()

ax = fig.add_subplot(1, 1, 1)

ax.tick_params(direction='in', length=5, width=1.5)

wl = 594

date = '20191005'

path = "C:\\Users\\elain\\Desktop\\data example\\paper data\\"+date

os.chdir(path)

nbin = 5

loc = [35,29,37]

x = []

y = []

for slidenum in range(0,len(loc)):

fnamer = str(slidenum+1)+"\\"+str(slidenum+1)+"_"+str(wl)+".txt"

txt = open(fnamer, "r")

for i in range(0,10000):

line = txt.readline().split('\t')

if(line[0] == ''):

txt.close()

break

if(float(line[0]) <= 1.5):

x.append(float(line[0]))

if(float(line[1]) >= 0.5):

y.append(float(line[1])-0.5)

if(float(line[1]) < 0.5):

y.append(abs(float(line[1])-0.5))

n,bint= np.histogram(y, bins = nbin, range = (0,0.5),density=1)

print(n,bint)

bins = np.array(n)

for i in range(0,nbin):

bins[i] = (bint[i]+bint[i+1])/2

plt.plot(bins,n,color = '#D6604D',label = 'mono',linewidth = 2)

print(len(x))

#plt.ylim(0,1.7)

#-------------------------------------------------------------------------------

wl = 594

date = '20191015'

path = "C:\\Users\\elain\\Desktop\\data example\\paper data\\"+date

os.chdir(path)

loc = [28]

x = []

y = []

for slidenum in range(0,len(loc)):

fnamer = str(slidenum+1)+"\\"+str(slidenum+1)+"_"+str(wl)+".txt"

txt = open(fnamer, "r")

for i in range(0,10000):

line = txt.readline().split('\t')

if(line[0] == ''):

txt.close()

break

if(float(line[0]) <= 1.5):

x.append(float(line[0]))

if(float(line[1]) >= 0.5):

y.append(float(line[1])-0.5)

if(float(line[1]) < 0.5):

y.append(abs(float(line[1])-0.5))

date = '20191008'

path = "C:\\Users\\elain\\Desktop\\data example\\paper data\\"+date

os.chdir(path)

loc = [40,35]

for slidenum in range(0,len(loc)):

fnamer = str(slidenum+1)+"\\"+str(slidenum+1)+"_"+str(wl)+".txt"

txt = open(fnamer, "r")

for i in range(0,10000):

line = txt.readline().split('\t')

if(line[0] == ''):

txt.close()

break

if(float(line[0]) <= 1.5):

x.append(float(line[0]))

if(float(line[1]) >= 0.5):

y.append(float(line[1])-0.5)

if(float(line[1]) < 0.5):

y.append(abs(float(line[1])-0.5))

n,bint= np.histogram(y, bins = nbin, range = (0,0.5),density=1)

print(n,bint)

bins = np.array(n)

for i in range(0,nbin):

bins[i] = (bint[i]+bint[i+1])/2

plt.plot(bins,n,color = '#4393C3',label = 'dual old',linewidth = 2)

print(len(x))

#------------------------------------------------------------------------------------

wl = 488

date = '20191015'

path = "C:\\Users\\elain\\Desktop\\data example\\paper data\\"+date

os.chdir(path)

loc = [28]

x = []

y = []

for slidenum in range(0,len(loc)):

fnamer = str(slidenum+1)+"\\"+str(slidenum+1)+"_"+str(wl)+".txt"

txt = open(fnamer, "r")

for i in range(0,10000):

line = txt.readline().split('\t')

if(line[0] == ''):

txt.close()

break

if(float(line[0]) <= 1.5):

x.append(float(line[0]))

if(float(line[1]) >= 0.5):

y.append(float(line[1])-0.5)

if(float(line[1]) < 0.5):

y.append(abs(float(line[1])-0.5))

date = '20191008'

path = "C:\\Users\\elain\\Desktop\\data example\\paper data\\"+date

os.chdir(path)

loc = [40,35]

for slidenum in range(0,len(loc)):

fnamer = str(slidenum+1)+"\\"+str(slidenum+1)+"_"+str(wl)+".txt"

txt = open(fnamer, "r")

for i in range(0,10000):

line = txt.readline().split('\t')

if(line[0] == ''):

txt.close()

break

if(float(line[0]) <= 1.5):

x.append(float(line[0]))

if(float(line[1]) >= 0.5):

y.append(float(line[1])-0.5)

if(float(line[1]) < 0.5):

y.append(abs(float(line[1])-0.5))

n,bint= np.histogram(y, bins = nbin, range = (0,0.5),density=1)

print(n,bint)

bins = np.array(n)

for i in range(0,nbin):

bins[i] = (bint[i]+bint[i+1])/2

plt.plot(bins,n,color = '#66C2A5',label = 'dual new',linewidth = 2)

print(len(x))

plt.legend(fontsize = 20)

plt.xlabel('$\mathdefault{N_y}$',fontsize = 25)

plt.ylabel('Probability density',fontsize = 25)

#plt.xticks(np.arange(0,0.7,0.2))

#plt.yticks(np.arange(1.5,,0.5))

plt.ylim(1.16,2.4)

plt.xlim(0,0.5)

plt.tight_layout()

plt.show()

- Simulation of BFM distribution evolution with 3 generations (Figure 4G)

import numpy as np

import matplotlib.pyplot as plt

from scipy.stats import truncnorm

import os

import random

from scipy.stats import norm,truncexpon,alpha,argus,rayleigh,beta,dweibull,trapz

import matplotlib as mpl

plt.rcParams["figure.figsize"] = [7,5.7]

mpl.rcParams['axes.linewidth'] = 1.5

mpl.rcParams['font.family'] = 'serif'

plt.rcParams['xtick.labelsize']=20

plt.rcParams['ytick.labelsize']=20

fig = plt.figure()

ax = fig.add_subplot(1, 1, 1)

ax.tick_params(direction='in', length=5, width=1.5)

#------------------------------------------------------------------------

def get_truncated_normal(mean=0, sd=1, low=0, upp=10):

return truncnorm((low - mean) / sd, (upp - mean) / sd, loc=mean, scale=sd)

path = "D:\\Sun\\motorsgroup\\data\\20190130"

#generation 0

n0 = 200000

rn = 7

sd = 0.4

mu = 0

nbin = int(1/0.05)

print(nbin)

gtn = get_truncated_normal(mean=mu, sd=sd, low=-0.5, upp=0.5)

y0 = gtn.rvs(size= n0)

#chi = 1

#y0 = rayleigh.rvs(size=n0)

#y0 = y0/5-0.5

#a, b = 2.31, 0.627

#y0 = beta.rvs(a,b, size=n0)

#y0 = y0-0.5

#c = 4

#y0 = dweibull.rvs(c, size=n0)

#y0 = y0/4

#c, d = 0.2, 0.8

#y0 = trapz.rvs(c, d, size=n0)

#y0 = y0-0.5

n,bint = np.histogram(y0, bins = nbin,density=1)

bins = np.array(n)

for i in range(0,nbin):

bins[i] = (bint[i]+bint[i+1])/2

plt.plot(bins,n,label = '0th generation',linewidth = 2,color = '#FFCC00')

#plt.title('0th generation - '+str(len(y0)))

print(np.max(y0),np.min(y0))

print(y0)

#iteration

yn = np.array(y0)

print(yn)

g = 3

for i in range(0,g):

for j in range(0,len(y0)):

if(y0[j] >= 0 and y0[j] < 0.5):

yn[j] = round(y0[j]*2 - 0.5,rn)

if(y0[j] < 0) and y0[j] > -0.5:

yn[j] = round(y0[j]*2 + 0.5,rn)

y0[j] = round(yn[j],rn)

yn = np.array(y0)

print(yn)

n,bint = np.histogram(yn, bins = nbin, range = (-0.5,0.5),density=1)

bins = np.array(n)

for j in range(0,nbin):

bins[j] = (bint[j]+bint[j+1])/2

if(i == 0):

plt.plot(bins,n,label = str(i+1)+'st generation',linewidth = 2,color = '#7BC043')

if(i == 1):

plt.plot(bins,n,label = str(i+1)+'nd generation',linewidth = 2,color = '#44B3C2')

if(i == 2):

plt.plot(bins,n,label = str(i+1)+'rd generation',linewidth = 2,color = '#32466F')

plt.xlim(-0.5,0.5)

#plt.ylim(0.55,0.5)

#plt.title(str(g)+'th generation - '+str(len(yn)))

plt.xticks(np.arange(-0.5,0.7,0.2))

plt.legend()

plt.xlabel('$\mathdefault{N_y}$',fontsize = 25)

plt.ylabel('Probability density',fontsize = 25)

plt.tight_layout()

plt.show()

#plt.savefig("C:\\Users\\motorsgroup\\Desktop\\figure\\5c.svg")

- Positioning stability (Figure S1)

import numpy as np

import scipy as sp

import matplotlib.pyplot as plt

import os

from scipy.optimize import curve_fit

import matplotlib as mpl

from cycler import cycler

from scipy.stats import norm

import matplotlib.mlab as mlab

import pandas as pd

from pandas.core.frame import DataFrame

plt.rcParams["figure.figsize"] = [7.5,3.5]

def f(x, a, b): # this is your 'straight line' y=f(x)

return a*x + b

mpl.rcParams['axes.linewidth'] = 1.5

label_size = 18

mpl.rcParams['xtick.labelsize'] = label_size

mpl.rcParams['ytick.labelsize'] = label_size

mpl.rcParams["font.family"] = 'serif'

mpl.rcParams['axes.prop_cycle'] = cycler('color', ['#D6604D'

,'#B31B2D'

,'#4393C3'

,'#2166AC'

,'#66C2A5'

,'#35978F'])

fs = 20

xtot = []

ytot = []

#fig = plt.figure(0)

#ax=fig.add_subplot(1,1,1)

#ax.tick_params(direction='in', length=6, width=1.5)

df = []

df.append(np.arange(0,11,1))

n0 = 0

for num in range(0,4):

if(num == 0):

date = "0525"

nnn = 5

nn = [2,0,2,1,2]

mm = [2,4,4,1,1,1,1]

if(num == 1):

date = "0525_2"

nnn = 5

nn = [2,1,2,1,2]

mm = [3,1,2,1,1,1,1,3]

if(num == 2):

date = "0525_3"

nnn = 4

nn = [0,2,1,2]

mm = [2,3,2,1,2]

if(num == 3):

date = "0525_4"

nnn = 4

nn = [2,2,2,1]

mm = [3,1,1,1,2,1,2]

path = "D:\\desktop\\Tracking ver.5\\"+date

os.chdir(path)

mnum = -1

npf = 1

for t in range(0,nnn):

for u in range(0,int(nn[t])):

mnum = mnum + 1

for ww in range(0,int(mm[mnum])):

fnamer = "y ad\\"+str(t+1)+"-"+str(u+1)+"_"+str(ww+1)+".txt"

txt = open(fnamer, "r")

yad = []

for j in range(0,25):

line = txt.readline().split('\t')

if (line[0] == ''):

txt.close()

break

yad.append(float(line[0]))

yad = np.array(yad)

yad = yad*1000

fnamer = "x ad\\"+str(t+1)+"-"+str(u+1)+"_"+str(ww+1)+".txt"

txt = open(fnamer, "r")

xad = []

for j in range(0,25):

line = txt.readline().split('\t')

if (line[0] == ''):

txt.close()

break

xad.append(float(line[0]))

xad = np.array(xad)

xad = xad*1000

for k in range(0,len(xad)):

xtot.append(xad[k]-np.mean(xad))

ytot.append(yad[k]-np.mean(yad))

n0 = n0 + 1

for k in range(0,len(yad)):

if(abs(yad[k]-np.mean(yad)) > 50):

print(date,str(t+1)+"-"+str(u+1)+"_"+str(ww+1))

df.append(xad - np.mean(xad))

df.append(yad - np.mean(yad))

'''

plt.subplot(2,1,1)

plt.title('Lateral',fontsize = 20)

plt.plot(xad - np.mean(xad))

plt.ylim(-300,300)

plt.xlim(0,10)

plt.tick_params(direction='in', length=6, width=1.5)

plt.ylabel('Position (nm)',fontsize = 20)

plt.xlabel('Time (min)',fontsize = 20)

plt.subplot(2,1,2)

plt.title('Axial',fontsize = 20)

plt.plot(yad - np.mean(yad))

plt.ylim(-300,300)

plt.xlim(0,10)

plt.tick_params(direction='in', length=6, width=1.5)

plt.ylabel('Position (nm)',fontsize = 20)

plt.xlabel('Time (min)',fontsize = 20)

'''

plt.subplot(1,2,1)

n, bins, _= plt.hist(xtot,bins = 15, normed=True)

plt.xlim(-300,300)

mu, sigma = norm.fit(xtot)

gua = mlab.normpdf( bins, mu, sigma)

plt.plot(bins, gua, 'r--')

plt.title('$\mathregular{σ_x}$ = '+str(round(sigma,1))+'nm',fontsize = 20)

plt.ylabel('Probability density',fontsize = 20)

plt.xlabel('Position (nm)',fontsize = 20)

plt.tick_params(direction='in', length=6, width=1.5)

print('x','mu = '+str(mu),'sigma = '+str(sigma))

plt.subplot(1,2,2)

n, bins, _= plt.hist(ytot,bins = 15, normed=True)

plt.xlim(-300,300)

mu, sigma = norm.fit(ytot)

gua = mlab.normpdf( bins, mu, sigma)

plt.plot(bins, gua, 'r--')

plt.ylabel('Probability density',fontsize = 20)

plt.xlabel('Position (nm)',fontsize = 20)

plt.title('$\mathregular{σ_y}$ = '+str(round(sigma,1))+'nm',fontsize = 20)

plt.tick_params(direction='in', length=6, width=1.5)

print('y','mu = '+str(mu),'sigma = '+str(sigma))

dff = DataFrame(df)

dff.to_csv('C:\\Users\\elain\\Desktop\\data example\\test.csv')

print(n0)

plt.tight_layout()

plt.show()
